# Effect of *Morchella esculenta* polysaccharides on the rectal microbiota of mice challenged with lipopolysaccharides

**DOI:** 10.3389/fvets.2024.1446924

**Published:** 2024-09-19

**Authors:** Yingjun Zhang, Reng Qiu, Zhifeng Zhang, Mikhlid H. Almutairi, Shah Nawaz, Shiqi Dong

**Affiliations:** ^1^College of Life Science, Nanyang Normal University, Nanyang, China; ^2^Department of Zoology, College of Science, King Saud University, Riyadh, Saudi Arabia; ^3^Department of Anatomy, Faculty of Veterinary Science, University of Agriculture, Faisalabad, Pakistan; ^4^Department of Veterinary Medicine, Southwest University, Chongqing, China

**Keywords:** *Morchella esculenta* polysaccharides, LPS, intestinal flora, rectal microbiota, intestinal dysfunction

## Abstract

**Introduction:**

Intestinal dysfunction poses a severe problem by preventing the digestion and absorption of nutrients. The gut, being the most vital organ for these processes, plays a crucial role in ensuring our body receives the nutrients it needs. We explored the mitigating effect of *Morchella esculenta* polysaccharides (MEP) on intestinal injury induced by lipopolysaccharides (LPS) through the modulation of intestinal flora.

**Methods:**

For this purpose, Kunming mice (KM) were divided into three groups, namely, PC, PM, and PY. Group PY was treated with MEP, while groups PM and PY were induced with LPS.

**Results:**

The results showed that weight loss in the PM group was significantly greater than that in the PY group (*P* < 0.05), and the organ indexes of the lung and spleen in the PM group were significantly higher than those in the PC (*P* < 0.01) and PY (*P* < 0.05) groups. LPS caused severe injuries in KM mice in the PM group, characterized by broken villi. However, MEP treatment could alleviate this damage in the PY group, resulting in relatively intact villi. The serum analysis showed that tumor necrosis factor alpha (TNF-ɑ) (*P* < 0.01), interleukin 6 (IL-6) (*P* < 0.01), and 3,4-methylenedioxyamphetamine (MDA) (*P* < 0.05) levels were significantly higher in the PM group, while IL-10 (*P* < 0.001), superoxide dismutase (SOD) (*P* < 0.01) and glutathione peroxidase (GSH-Px) (*P* < 0.01) were significantly lower in that group. Interestingly, supplementation with MEP could lower the levels of TNF-ɑ, IL-10, IL-6, MDA while increasing the levels of superoxide dismutase (SOD) (*P* < 0.01) and GSH-Px. The gut microbiota analysis yielded 630,323 raw reads and 554,062 clean reads, identifying 3,390 amplicon sequencing variants (ASVs). One phylum and five genera were notably different among animal groups, including *Escherichia_Shigella, Limosilactobacillus*, unclassified_*Geminicoccaceae*, unclassified_*Rhodobacteraceae*, and *Parabacteroides* (*P. distasonis*).

**Discussion:**

In conclusion, we found that MEP could mitigate the intestinal damage caused by LPS by modulating the inflammatory response, oxidative resistance, and intestinal flora of KM mice. Our results may provide insights into novel treatment options for intestine-related diseases.

## Introduction

The gut is the most important organ for the digestive absorption of nutrients. Various enzymes in the villi contribute to digestion, while intestinal epithelial cells combined with tight junction proteins promote absorption. Intestinal integrity is crucial for host health, and compromised intestines have been reported in individuals with colitis as well as those affected by infectious bacterial or viral intestinal diseases (1, 2). Gut flora refers to the microorganisms inhabiting the intestines, including commonly known bacteria, viruses, and fungi, as well as millions of archaea and protozoa (3, 4). In individuals, the intestinal microbiota is positively related to the host's nutrition and xenobiotic metabolism, immunity, and body homeostasis (3, 5, 6). Intestinal epithelial cells (IECs) and the intestinal microbiota function together as a protective barrier against toxins and pathogens (7). An imbalance in the structure of gut flora may lead to metabolic disorders in the host (8), and dysbiosis is commonly observed in various diseases, including lung disease, kidney disease, and diarrhea (9–11). There is an urgent need for novel and effective therapies for treating gastrointestinal diseases.

The well-known endotoxin lipopolysaccharide is distributed in the external panniculus of Gram-negative microbes, guaranteeing its bacterial structure and function integrity (12). The bacteria release LPS after multiplication, death, and lysis. The released lipopolysaccharides (LPS) cause not only an inflammatory response but also fever, septic shock, diarrhea, organ damage, and even serious physiological effects (13, 14). Among these complications, sepsis is a common disease with high mortality, which has limited therapeutic options for its treatment (13). Additionally, dysbiosis caused by LPS has been reported in several investigations (15–17). Polysaccharides consist of numerous polymer carbohydrates that have important biological functions, such antioxidant effects, antitumor activities, anti-microbial properties, and the ability to regulate the immune system (18, 19). *Morchella esculenta (L.) Pers* is a well-known edible and medicinal fungus, which is recorded in the Chinese medicine masterpiece *Compendium of Materia Medica* (also known as Pen-tsao Kan-mu) as having the effect of “benefiting the intestines and stomach, helping food digestion and absorption”. Polysaccharides from *Morchella esculenta (L.) Pers* (MEP) are highly valued healthcare products known for their biological activities, including oxidation resistance (20), anti-inflammatory effects, and regulatory flora properties (43). However, there is only a limited understanding regarding the effect of MEP on mice challenged with LPS. Hence, we performed this trial to understand the remission effect of MEP on animals induced by LPS through the modulation of intestinal flora.

## Materials and methods

### Animals

A total of 36 Kunming (KM) mice, aged 4 weeks (21.50 ± 0.67 g), with equal numbers of male and female animals were used in this study. They were obtained from the Experimental Animal Center at Yangzhou University (Yangzhou, China). All KM mice were divided into three groups, namely PC (the control group), PM (the model group), and PY (the MEP group), after acclimatization for 3 days. The mice in the PY group were supplemented with MEP (100 mg/kg) via intragastric administration for 14 days, while KM mice in the other groups were given the same volume of normal saline. On the 15th day, the KM mice in the PM and PY groups were intraperitoneally induced with LPS (10 mg/kg, Acmec Biochemical Technology Co., Ltd., Shanghai, China). Then, all KM mice were euthanized the following day to collect serum, organs, and large intestines (21). The daily weight of KM mice and organs was documented and the organ index was calculated.

### Extraction and purification of MEP

First, 500 g of *Morchella esculenta (L.) Pers* was decolored and defatted with 95% ethanol (2,000 mL) twice under reflux for 1 h each time. After reflux, ethanol was removed, and the residue was dried at 60°C. The dried residue was extracted by boiling water (4,000 mL) twice for 1.5 h each time. Finally, the extract was filtered, combined, and concentrated. After cooling to room temperature, the concentration was adjusted to 500 mL by deionized water and then 90% ethanol (v/v) was added until the total percentage of ethanol in the solution reached 80%. The mixture was kept at 4°C overnight and then filtered to give the total crude *Morchella esculenta (L.) Pers* polysaccharides (MEPct). The MEPct solution (0.1 g/mL) was prepared using deionized water, and the proteins in the MEPct solution were sequentially removed five times using the Sevag method. The deproteined solution was concentrated to 500 mL under vacuum and then freeze-dried at −20°C for 1 week to obtain the total purified MEP.

### Histopathological analysis

Large intestine tissues obtained from KM mice were fixed in paraformaldehyde for further hematoxylin and eosin (H&E) staining (Pinuofei Biological, Wuhan, China). Subsequently, the pathological examination was carried out using a microscope (Olympus BX46, Japan) following the methodology of a previous study (22).

### Antioxidant ability and inflammatory factor in KM mice

Serum samples obtained from KM mice were tested for indexes of antioxidant abilities and inflammatory factors [tumor necrosis factor alpha (TNF-ɑ), interleukin 6 (IL-6), IL-10, IL-1β, superoxide dismutase (SOD), total antioxidant capacity (T-AOC), methylenedioxyamphetamine (MDA), and glutathione peroxidase (GSH-Px)] using assay kits provided by the Jiancheng Bioengineering Research Institute and Solarbio Life Science, following the previously described methods (23).

### Rectal flora sequencing and analysis

A total of nine rectum samples from KM mice were used for DNA extraction, and amplification of the V3–V4 variable region of the 16S rRNA gene was conducted (24). The generated products were then processed for library construction using the Hieff NGS Ultima Pro DNA Library Prep Kit (Yeasen, China) and sequenced via the Bioyi Biotechnology Illumina NovaSeq platform (Wuhan, China). The sequenced raw data were subjected to quality control via Trimmomatic (v0.33) (25), Cutadapt (1.9.1) (26), and QIIME2 (27). Then, the filtered data were used for amplicon sequence variant (ASV) analysis (28) and taxonomy annotation.

### Statistical analysis

Data regarding KM mice were presented as means ± standard deviation and analyzed using SPSS version 26.0. Alpha and beta diversity analyses were performed by employing QIIME (29, 30). Significant differences among the three KM animal groups were determined by analysis of variance (ANOVA), linear discriminant analysis (30), and Metastats (31). The network analysis of the intestinal flora in KM mice was performed using Spearman's rank correlation analysis (32). A *p*-value of < 0.05 was considered statistically significant.

## Results

### The effect of MEP on weight and organ indexes in KM mice induced by LPS

**Figure 1:**
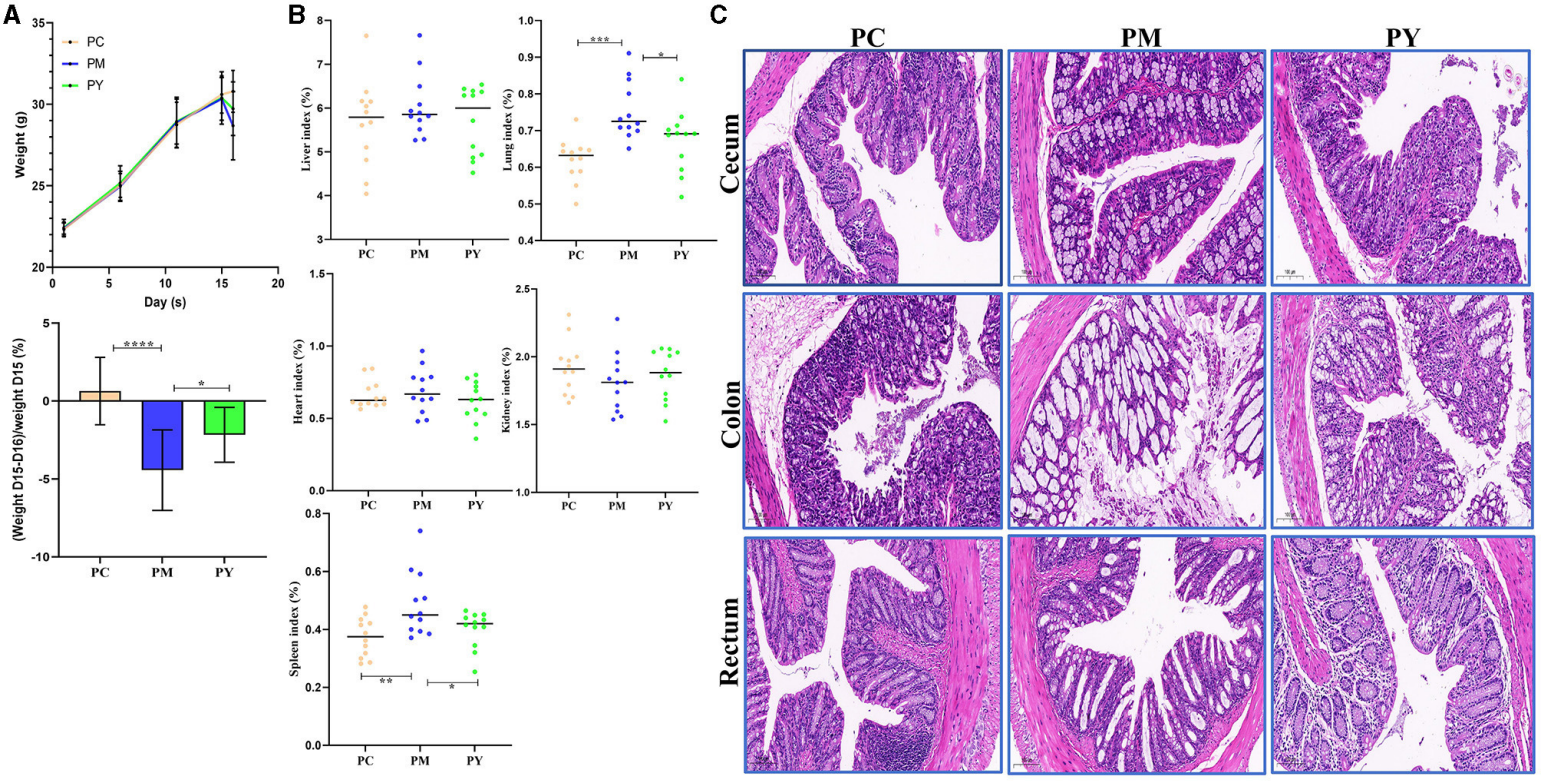
MEP-remitted damages in KM mice induced by LPS. **(A)** Body weight, **(B)** organ index, **(C)** pathological analysis. Scale bar 50 μm. Data are shown as mean ± SEM (*n* = 12). Significance is marked as *P < 0.05, **P < 0.01, ***P < 0.001, and ****P < 0.0001.

Although the mean body weight of the KM mice was nearly the same in the three groups, the weight loss in PM mice was dramatically higher than that in PY mice (*P* < 0.05) (Figure 1A). The organ indexes of the lung and spleen of KM mice in the PM group were higher than those in the PC (*P* < 0.01) and PY (*P* < 0.05) groups (Figure 1B). LPS caused severe injuries in KM mice in the PM group, characterized by broken villi, while MEP could alleviate this damage in the PY group with relatively intact villi (Figure 1C).

### The effect of MEP on oxidation resistance and inflammatory response in KM mice induced by LPS

**Figure 2:**
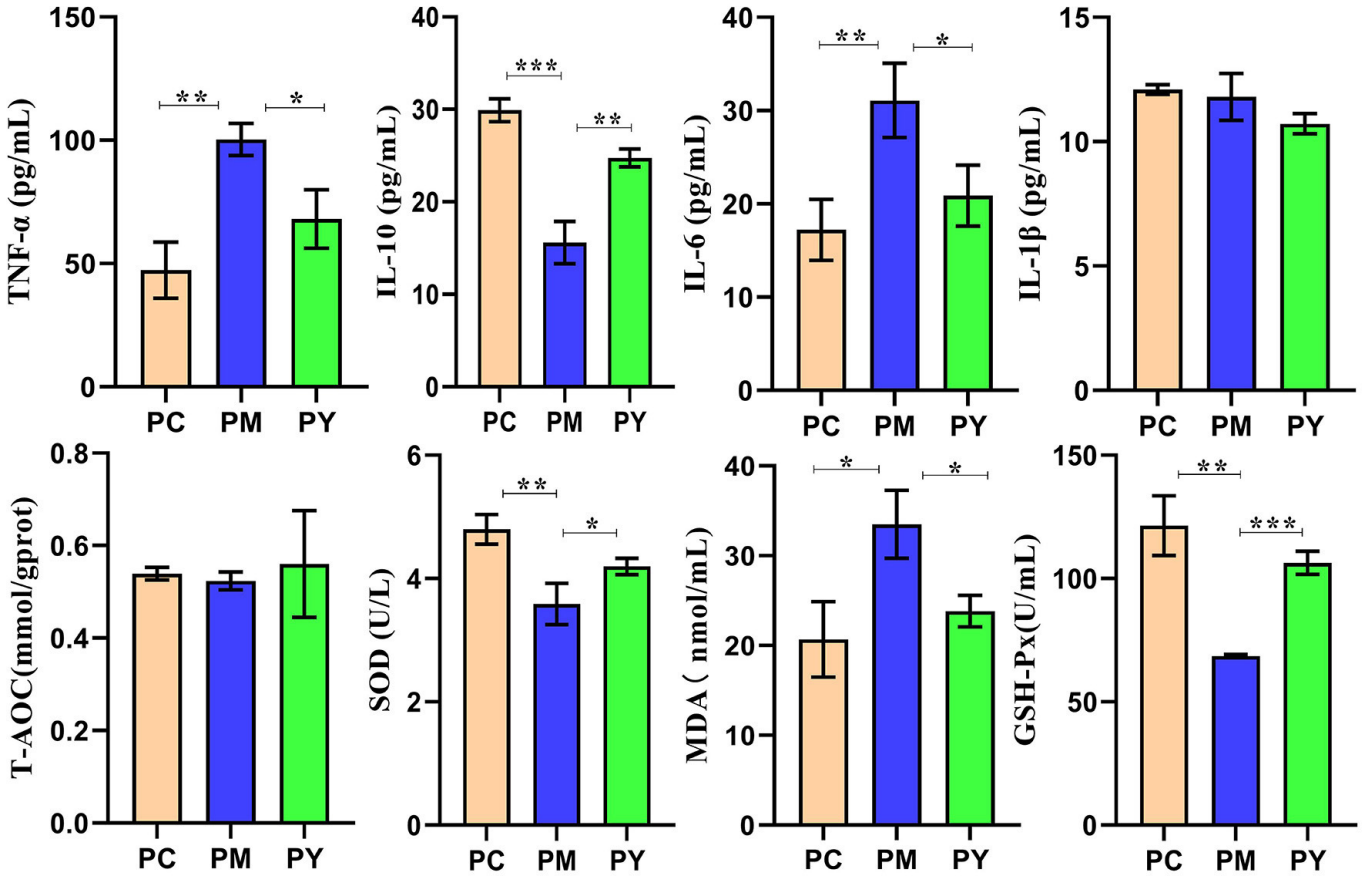
MEP promoted oxidation resistance and decreased inflammatory response in KM mice challenged by LPS. Data are exhibited as mean ± SEM (*n* = 3). Significance is marked as *P < 0.05, **P < 0.01, and ***P < 0.001.

Serum analysis showed that TNF-ɑ (*P* < 0.01), IL-6 (*P* < 0.01) and MDA (*P* < 0.05) were significantly higher in PM mice, while IL-10 (*P* < 0.001), SOD (*P* < 0.01), and GSH-Px (*P* < 0.01) were markedly lower in that group. Interestingly, supplementation with MEP decreased TNF-ɑ, IL-10, IL-6, and MDA levels and increased SOD (*P* < 0.01) and GSH-Px levels (Figure 2).

### The effect of MEP on gut flora of KM mice induced by LPS

**Figure 3:**
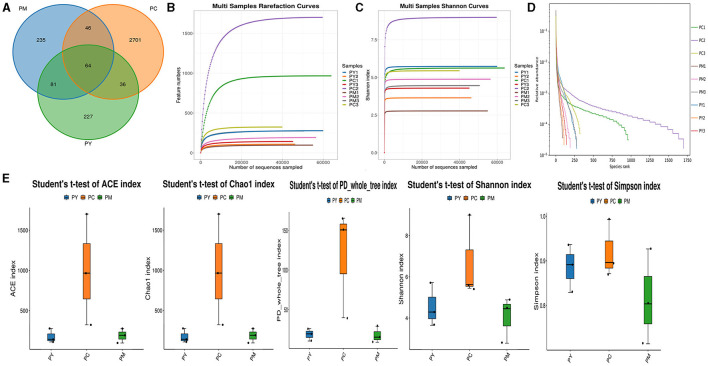
Intestinal flora structure and α-diversity analysis. **(A)** Venn map, **(B)** rarefaction curve, **(C)** Shannon index curve, **(D)** rank abundance curve, and **(E)** Statistical analysis of α-diversity.

Over 47,870 raw and 41,987 filtered reads were found in each sample of KM mice (Table 1), with 3,390 ASVs found in the three animal groups (Figure 3A). The curves of Shannon index, rarefaction, and rank abundance in KM mice were flat and displayed saturated curves (Figures 3B–D), demonstrating that the sequencing depth was sufficient in the current experiment. No marked difference in the α-diversity was observed between the groups (Table 2, Figure 3E).

**Table 1:** Sequencing data information.

**Samples**	**Raw reads**	**Clean reads**	**Denoised reads**	**Merged reads**	**Non-chimeric reads**
PC1	79,933	71,123	69,314	66,962	63,917
PC2	79,962	71,502	68,338	63,303	59,771
PC3	47,870	41,987	41,462	40,583	39,887
PM1	70,719	62,028	61,760	61,062	54,949
PM2	81,344	70,770	70,173	68,215	56,271
PM3	61,959	54,720	54,156	52,671	50,582
PY1	82,622	72,114	71,478	69,460	59,850
PY2	61,712	54,124	53,834	52,955	46,047
PY3	64,202	56,255	55,919	54,734	45,098

**Table 2:** Alpha diversity information of the microbiota of current mice.

**Sample**	**Feature**	**ACE**	**Chao1**	**Simpson**	**Shannon**	**PD_whole_tree**	**Coverage**
PC1	966	966.0	966.0	0.8711	5.6246	165.6135	1.0
PC2	1,700	1,700.1759	1,700.0	0.9926	8.9873	150.7546	1.0
PC3	324	324.0	324.0	0.8964	5.4438	39.7817	1.0
PM1	97	97.0	97.0	0.714	2.775	8.8589	1.0
PM2	192	192.7376	192.0588	0.9267	4.8809	15.2373	1.0
PM3	275	275.294	275.0	0.8036	4.4539	29.0528	1.0
PY1	278	278.5265	278.0667	0.9368	5.7317	26.1002	1.0
PY2	109	109.2779	109.0	0.8292	3.6431	10.3847	1.0
PY3	142	142.2855	142.0	0.8914	4.2884	19.415	1.0

**Figure 4:**
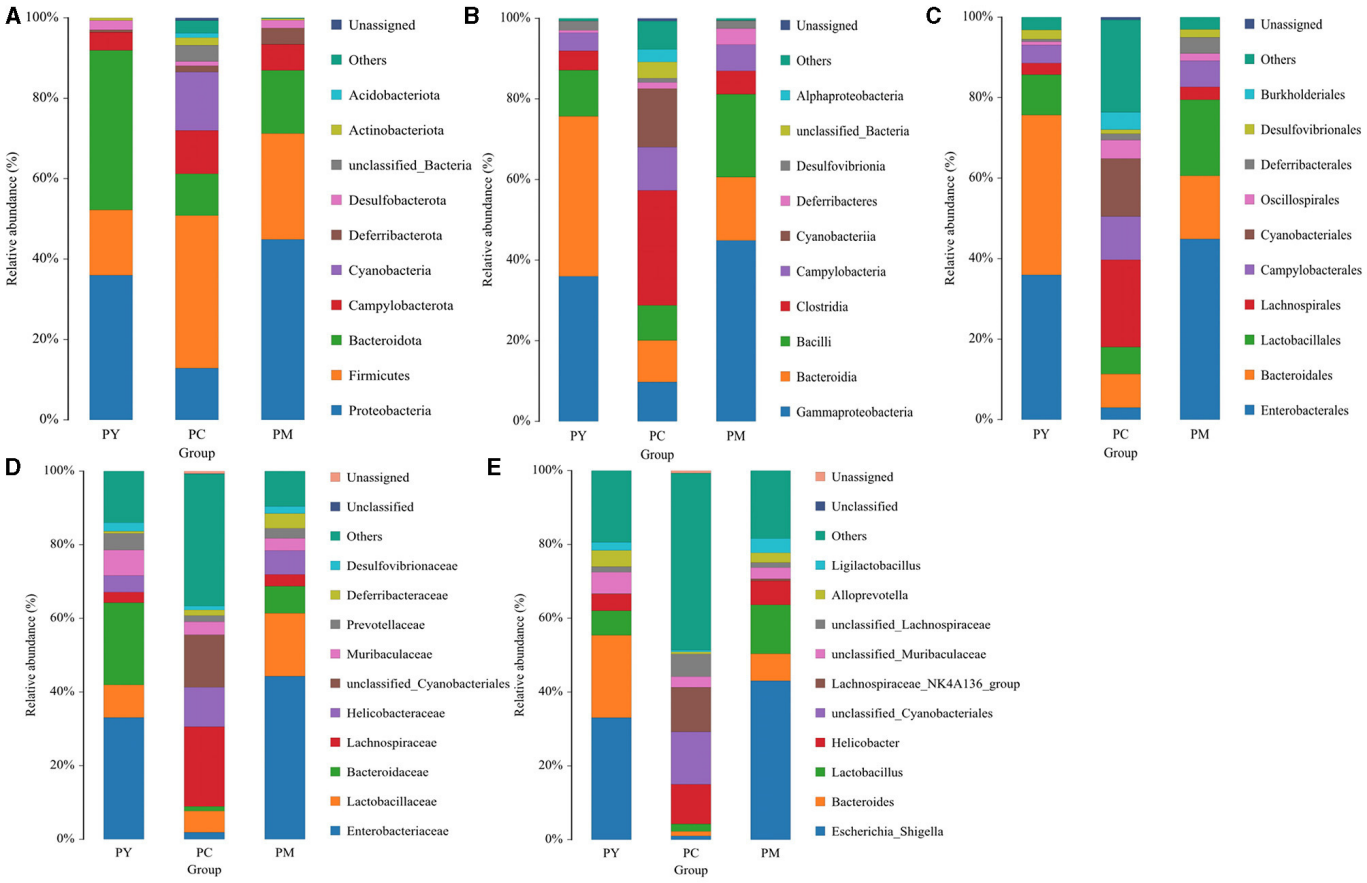
Comparing intestinal flora of KM mice in different taxa: **(A)** phylum, **(B)** class, **(C)** order, **(D)** family, and **(E)** genera.

**Figure 5:**
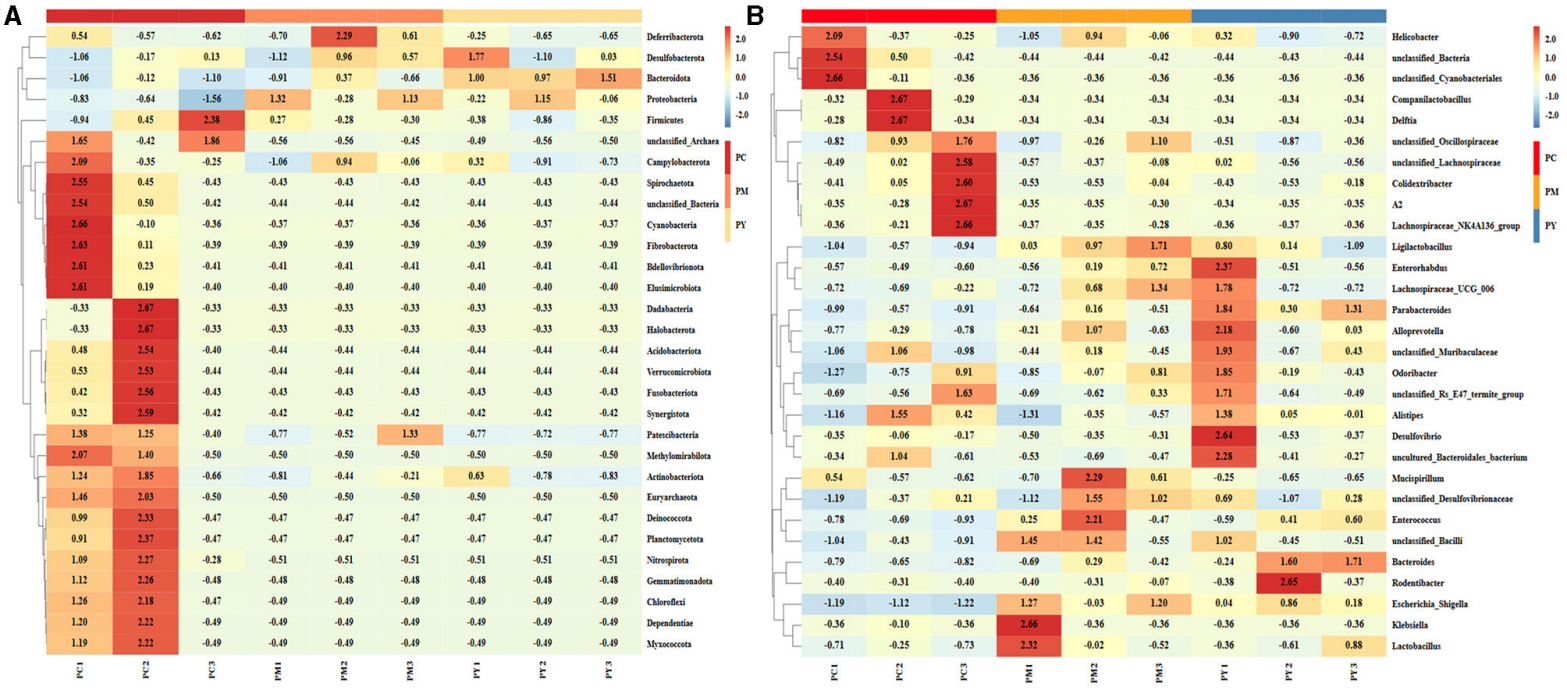
Heat map analysis of intestinal flora in different taxa: **(A)** phylum and **(B)** genera.

The predominant phyla in different groups were as follows: Firmicutes (phylum Firmicutes) (37.97%), Cyanobacteria (14.54%), and Proteobacteria (12.85%) in the PC group; Proteobacteria (44.88%), Firmicutes (26.36%), and Bacteroidota (15.71%) in the PM group; and Bacteroidota (39.70%), Proteobacteria (35.98%), and Firmicutes (16.24%) in the PY group (Figure 4A). At the class level, Clostridia (28.51%), Cyanophyceae (14.50%), and Bacteroidia (10.32%) were mainly observed in the PC group, while Gammaproteobacteria, Bacilli (*Bacillus subtilis*), and Bacteroidia were predominantly found in the PM (44.88, 20.58, and 15.71%) and PY (35.98, 39.70, and 11.45%) groups (Figure 4B). At the order level, Lachnospirales (21.67%), Cyanobacteriales (14.36%), and Campylobacterales (10.77%) were predominantly observed in the PC group, while Enterobacterales, Lactobacillales, and Bacteroidales were the dominant orders in the PM (44.87, 18.90, and 15.71%) and PY (35.97, 39.70, and 10.03%) groups (Figure 4C). At the family level, Lachnospiraceae (21.65%), unclassified_Cyanobacteriales (14.24%), and Helicobacteraceae (10.72%) were primarily dominant in the PC group, while Enterobacteriaceae, Lactobacillaceae, and Bacteroidaceae were the main families in the PM (44.30, 17.10, 7.35%) and PY (33.07, 8.86, 22.32%) groups (Figure 4D). At the genus level, unclassified_*Cyanobacteriales* (14.24%), *Lachnospiraceae*_NK4A136_group (12.04%), and *Helicobacter* (10.72%) were predominantly found in the PC group, while *Escherichia_Shigella, Lactobacillus*, and *Bacteroides* were the main genera in the PM (43.04, 13.23, and 7.34%) and PY (33.07, 6.64, and 22.32%) groups (Figure 4E). The heat map showed a higher abundance of phyla of unclassified_Archaea, Acidobacteriota, Nitrospirota, and Chloroflexi in the PC group, Proteobacteria in the PM group, and Bacteroidota in the PY group (Figure 5A). At the genus level, a higher abundance of *Lachnospiraceae*_NK4A136_group in the PC group, *Ligilactobacillus* and *Escherichia_Shigella* in the PM group, and *Parabacteroides, Bacteroides*, and *Escherichia_Shigella* in the PY group were observed (Figure 4B). The Krona analysis indicated that the main genera in each group were as follows: unclassified_*Cyanobacteriales*, unclassified_ *Lachnospiraceae*_NK4A136_group, unclassified_*Lachnospiraceae*, and unclassified_*Helicobacter* in the PC group; unclassified_*Escherichia_Shigella*, unclassified_*Lactobacillus*, and unclassified_*Bacteroides* in the PM group; and unclassified_*Escherichia_Shigella*, unclassified_Muribaculaceae, and unclassified_*Lactobacillus* in the PY group, respectively (Figure 5).

### Beta diversity analysis of intestinal flora

**Figure 6:**
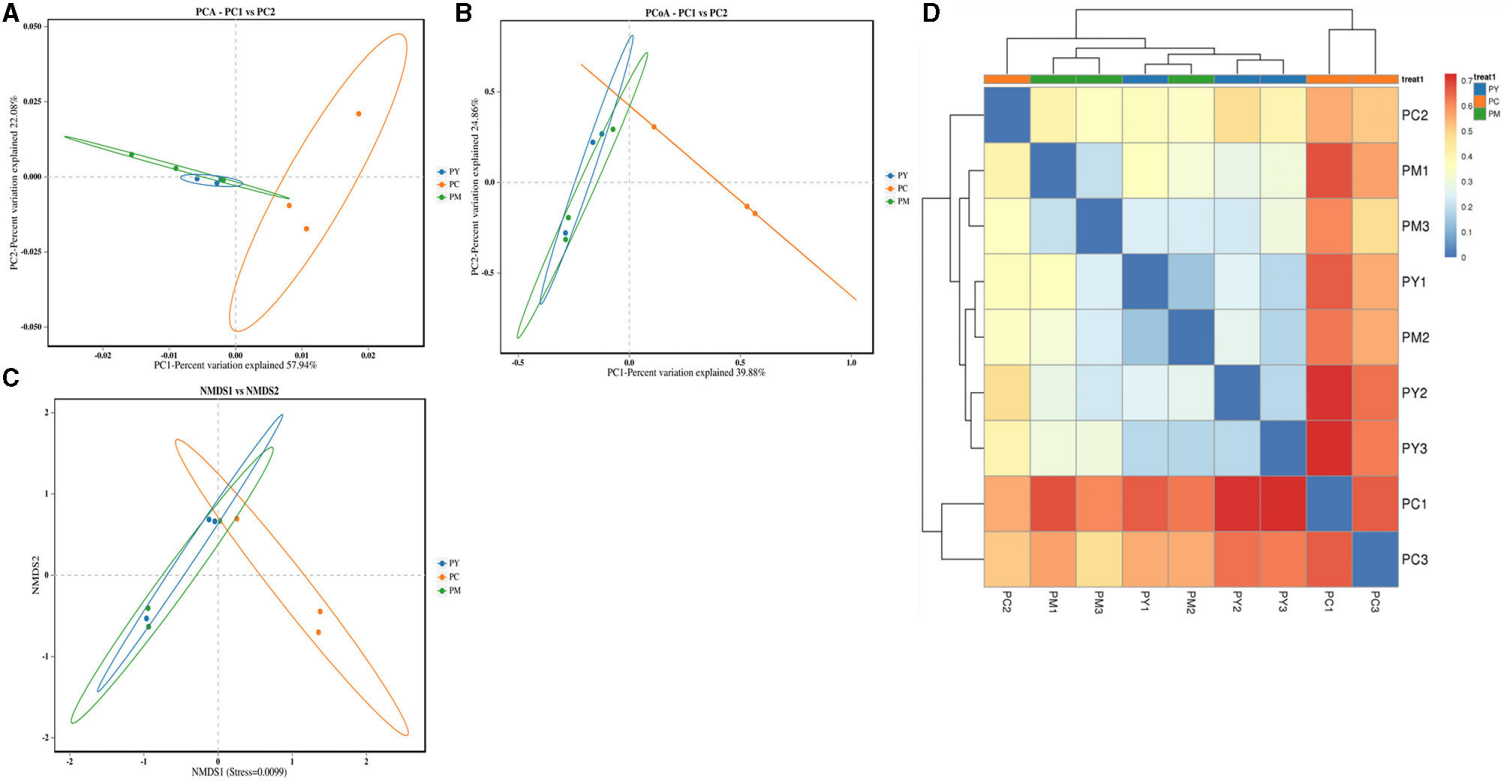
Beta diversity analysis of intestinal flora: **(A)** PCA, **(B)** PCoA, **(C)** NMDS, and **(D)** clustering heat map.

**Figure 7:**
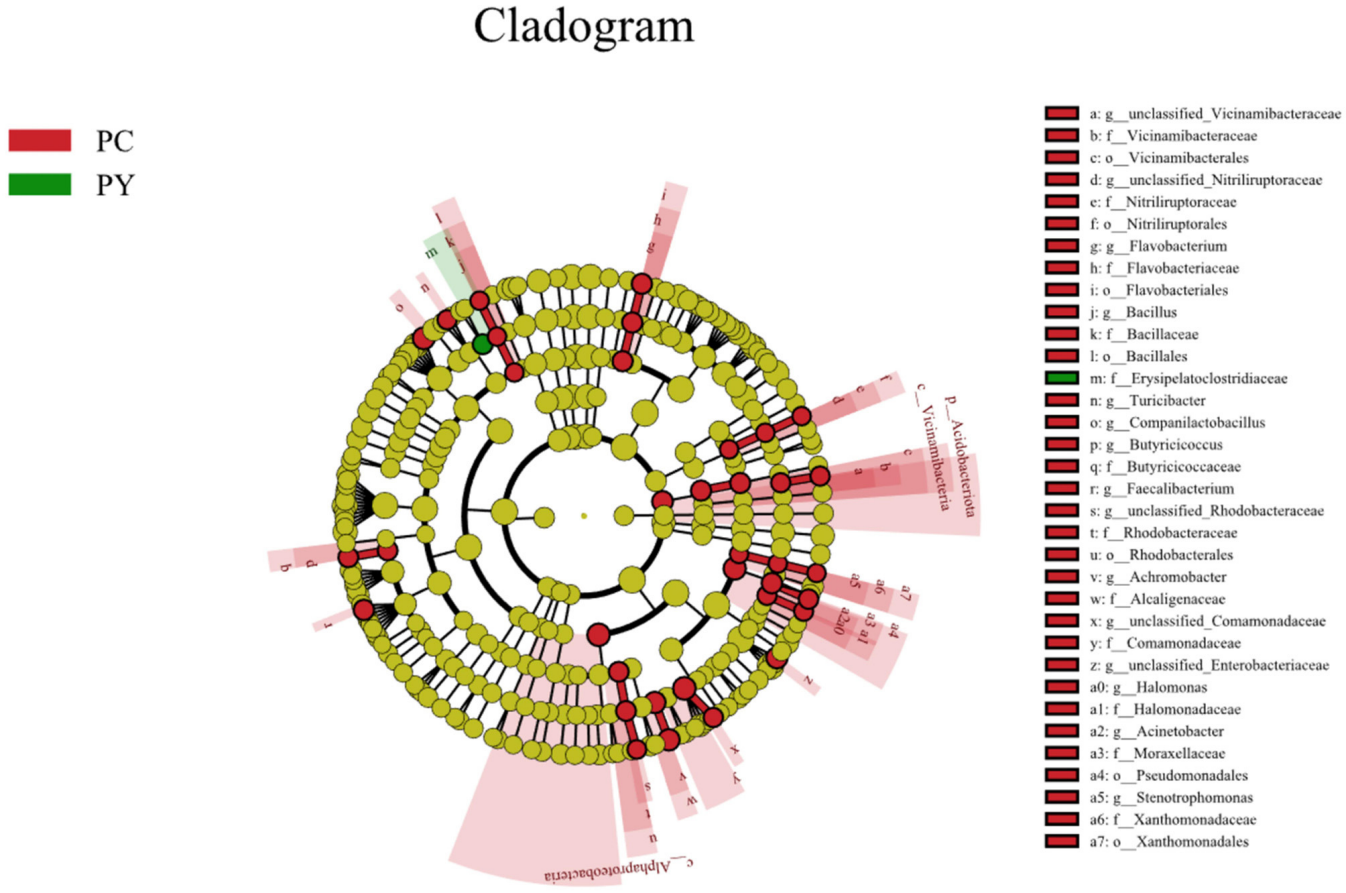
LEfSe analysis of intestinal flora.

**Figure 8:**
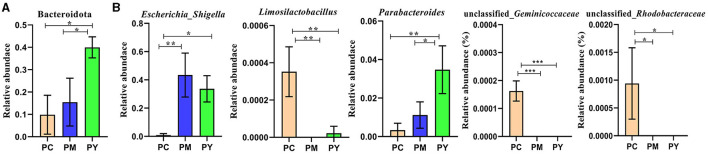
ANOVA of intestinal flora: **(A)** phylum and **(B)** genera. Significance is presented as *p < 0.05, **p < 0.01, and ***p < 0.001; data are presented as mean ± SEM (*n* = 3).

Beta diversity analysis showed that LPS-challenged KM mice were significantly distant from PC mice. However, the distance between PY and PC mice was shorter than that between PM and PC mice (Figure 6). The linear discriminant analysis effect size (LEfSe) revealed that taxa o_*Pseudomonadales* (*P* < 0.05), f_*Moraxellaceae* (*P* < 0.05), f_*Butyricicoccaceae* (*P* < 0.05), g_*Butyricicoccus* (*P* < 0.05), f_*Alcaligenaceae* (*P* < 0.05), g_*Achromobacter* (*P* < 0.05), g_unclassified_*Enterobacteriaceae* (*P* < 0.05), p_*Acidobacteriota* (*P* < 0.05), c_*Vicinamibacteria* (*P* < 0.05), o_*Vicinamibacterales* (*P* < 0.05), f_*Vicinamibacteraceae* (*P* < 0.05), g_unclassified_*Vicinamibacteraceae* (*P* < 0.05), o_*Nitriliruptorales* (*P* < 0.05), f_*Nitriliruptoraceae* (*P* < 0.05), g_unclassified_*Nitriliruptoraceae* (*P* < 0.05), o_*Flavobacteriales* (*P* < 0.05), f_*Flavobacteriaceae* (*P* < 0.05), g_*Flavobacterium* (*P* < 0.05), o_*Bacillales* (*P* < 0.05), f_*Bacillaceae* (*P* < 0.05), g_Bacillus (*P* < 0.05), g_*Turicibacter* (*P* < 0.05), g_*Companilactobacillus* (*P* < 0.05), g_*Faecalibacterium* (*P* < 0.05), c_*Alphaproteobacteria* (*P* < 0.05), o_*Rhodobacterales* (*P* < 0.05), f_*Rhodobacteraceae* (*P* < 0.05), g_unclassified_*Rhodobacteraceae* (*P* < 0.05), f_*Comamonadaceae* (*P* < 0.05), g_unclassified_*Comamonadaceae* (*P* < 0.05), f_*Halomonadaceae* (*P* < 0.05), g_*Halomonas* (*P* < 0.05), g_*Acinetobacter* (*P* < 0.05), o_*Xanthomonadales* (*P* < 0.05), f_*Xanthomonadaceae* (*P* < 0.05), and g_*Stenotrophomonas* (*P* < 0.05) were higher in the PC group, while f_*Erysipelatoclostridiaceae* (*P* < 0.05) was higher in the PY group (Figure 7). An ANOVA showed that *Bacteroidota* was significantly higher in the PY group (*P* < 0.05) (Figure 8A). At the genus level, the abundance of *Escherichia_Shigella* in the PC group was markedly lower compared to the PM (*P* < 0.01) and PY (*P* < 0.05) groups. *Limosilactobacillus* (*P* < 0.01), unclassified_*Geminicoccaceae* (*P* < 0.0001), and unclassified_*Rhodobacteraceae* (*P* < 0.05) were significantly higher in the PC group. *Parabacteroides* in the PY group was significantly higher compared to the PM (*P* < 0.05) and PC (*P* < 0.01) groups (Figure 8).

### Network and function analysis of intestinal flora

**Figure 9:**
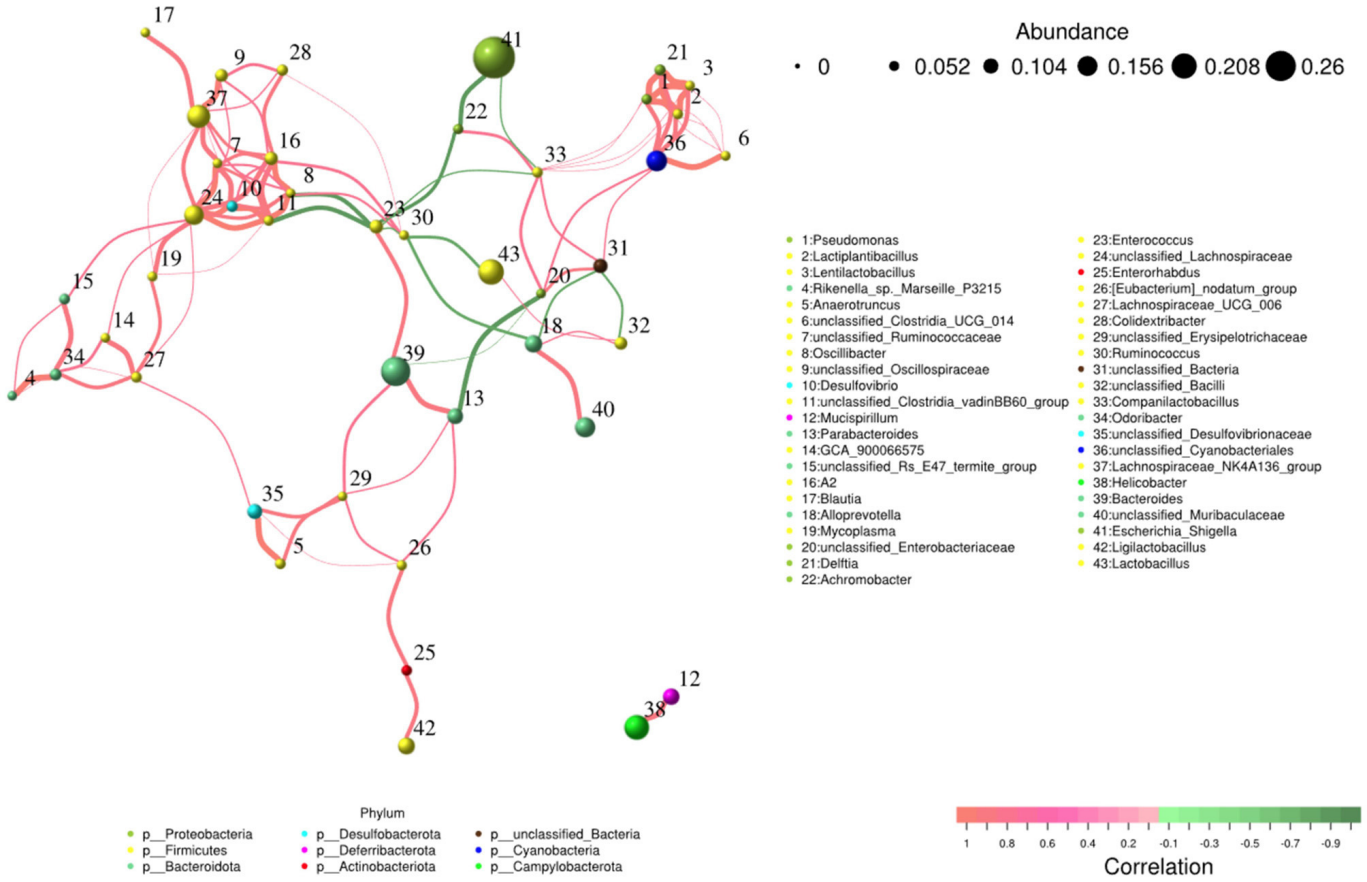
Network analysis of intestinal flora.

The network analysis revealed that *Escherichia_Shigella* genus negatively contributed to the gut flora, while *Lachnospiraceae*_NK4A136_group, *Bacteroides*, unclassified_*Lachnospiraceae*, and unclassified_*Cyanobacteriales* genera made positive contributions to the gut flora (Figure 9).

## Discussion

Plant and microorganism extracts are natural products with various biological activities such as immunoregulation, anti-inflammatory effects, oxidative stress reduction, and liver protection (33). In this study, we found that MEP could alleviate body weight loss in KM mice induced by LPS by maintaining the gut integrity (Figure 1). Consistent with previous studies reporting splenomegaly in animals treated with LPS (34, 35), a higher spleen index was observed in the PM group. However, MEP significantly decreased it (*P* < 0.05) (Figure 1B). Previous studies have also found an association between splenomegaly and IL-6 and TNF-α (35). Therefore, we examined the levels of inflammatory factors in the serum of KM mice. Similar to previous studies demonstrating higher levels of TNF-α and IL-6 and lower levels of IL-10 in LPS-induced animals (36, 37), we observed comparable results for these pro-inflammatory and anti-inflammatory cytokines in this study. Interestingly, MEP significantly reduced inflammation by notably decreasing TNF-α and IL-6 levels and increasing IL-10 levels in PY mice. The antioxidant system and reactive oxygen species are the two side effects of host health and their imbalance is associated with the disease (38). Oxidative damage is a well-known consequence of LPS exposure (39, 40), and SOD, GSH-Px, and MDA are indicators affected by LPS (16). Higher levels of MDA and lower levels of SOD and GSH-Px in PM animals were consistent with previous results (16, 40). However, MEP could reduce oxidative damage by regulating these enzymes in the PY group (Figure 2).

**Figure 10:**
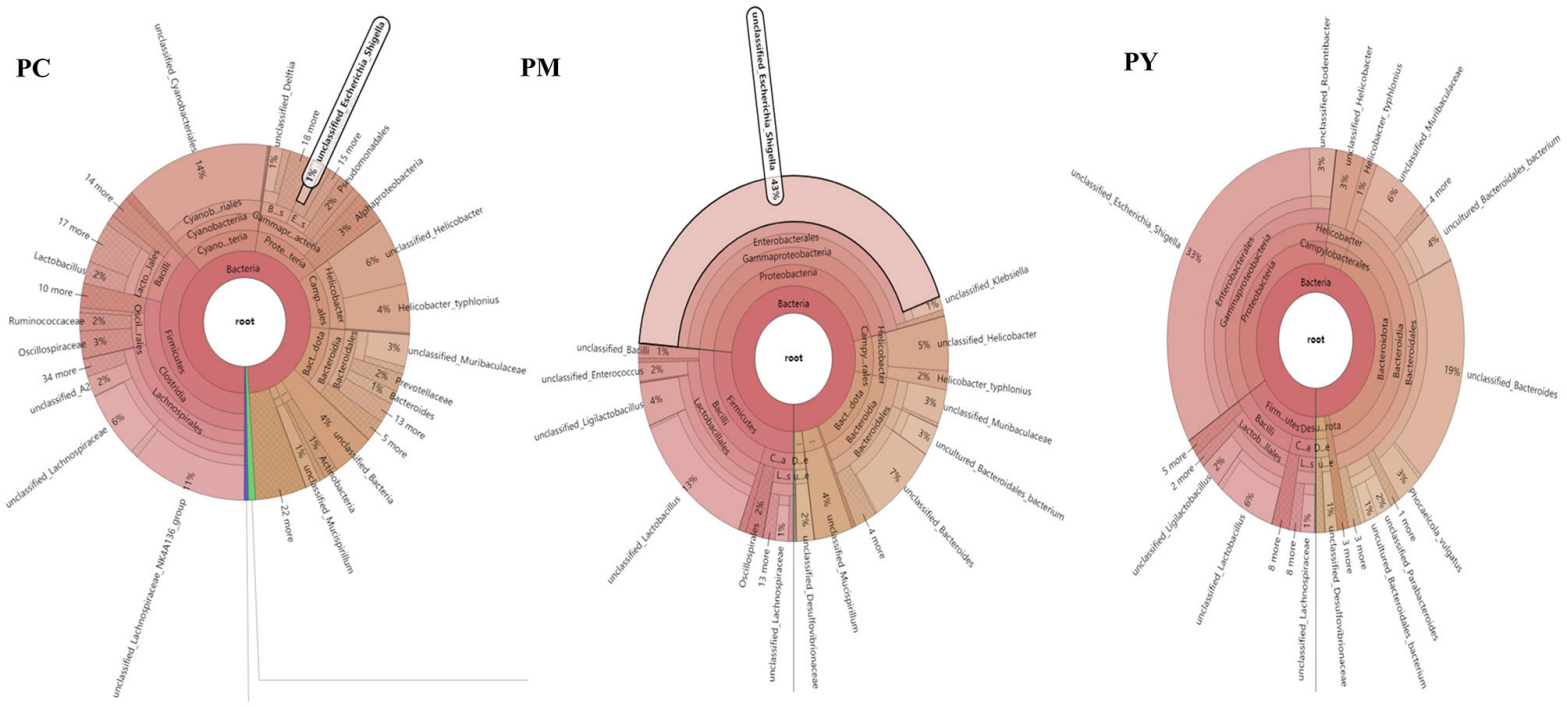
Krona analysis of intestinal flora.

In addition to inflammatory responses and oxidative injuries, dysbiosis was also observed in animals challenged with LPS (15, 16, 23). Next, we performed the gut microbiota analysis of KM mice and obtained 630,323 raw reads and 55,462 clean reads. These reads were identified with 3,390 ASVs, and 63 ASVs were shared among the KM mouse groups (Figure 3). No marked difference in α-diversity was observed between the KM groups, which is consistent with the findings in people receiving synbiotics (24) and liver-damaged mice (23). However, this is contrary to the observations in antibiotic-treated mice (21) and obese animals (44). LPS altered the abundance of bacteria across different taxa, and MEP were able to partially restore the microbiota structure in KM mice (Figures 4, 5, 10). In a healthy host, the primary phyla of microbiota are Firmicutes and Bacteroidetes (3). In this study, MEP increase the abundance of those two phyla in the PY group (55.96%) compared with the PM group (42.06%). Similar results were found at the genus level; LPS increase the abundance of *Escherichia_Shigella* (43.04%), while KM mice treated with MEP had a lower abundance of this genus. The proteobacteria *Escherichia Shigella* is a conditioned pathogen leading to intestine diseases (45). Furthermore, we explored the significant variations in phyla and genera among KM groups, detecting one phylum and five genera (Figure 8). These genera were *Escherichia_Shigella, Limosilactobacillus*, unclassified_*Geminicoccaceae*, unclassified_*Rhodobacteraceae*, and *Parabacteroides*. The network analysis revealed that *Escherichia_Shigella* was an important genus that negatively interacted with the gut flora (Figure 9). Species of *Limosilactobacillus* have been commercialized for use in probiotics (41), and a previous study found a lower abundance of *Limosilactobacillus* in heat-stressed birds (42). Interestingly, KM mice supplemented with MEP increased the abundance of this genus. These results suggest that MEP could regulate the intestinal microbiota in KM mice. However, due to species and strain differences, all the results and findings in this study require further research on other animal strains and species.

## Conclusion

In conclusion, our study demonstrates that MEP have the potential to alleviate intestinal damage induced by LPS in KM mice. By modulating inflammatory responses, enhancing oxidation resistance, and restoring intestinal flora, MEP show promise as a therapeutic agent in mitigating intestinal-related diseases. These findings offer valuable insights into the development of novel treatment options aimed at preserving gut health and combating the detrimental effects of LPS-induced damage.

## Data Availability

All raw data from ICR animals was deposited in the NCBI Sequence Read Archive under accession number: PRJNA1073062.
